# Association between domain-specific physical activity and mental health status after embryo transfer in IVF-ET-assisted pregnancy patients

**DOI:** 10.1038/s41598-024-55097-3

**Published:** 2024-02-28

**Authors:** Wei Wang, Fang Yang, Yunfeng Bai, Yanxia Lu, Xueqin Mao

**Affiliations:** 1 Department of Psychology, Qilu Hospital of Shandong University, Wenhuaxi Road 107, Jinan, 250012 Shandong China; 2 Center for Reproductive Medicine, Department of Obstetrics and Gynecology, Qilu Hospital of Shandong University, Jinan, China; 3 Department of Anesthesiology, Linyi People’s Hospital, Linyi, China; 4 Department of Medical Psychology and Ethics, School of Basic Medical Sciences, Cheeloo College of Medicine, Shandong University, Jinan, 250012 Shandong China

**Keywords:** Physical activity, Domain-specific, Activity intensity, Infertility, Mental health, Psychology, Human behaviour

## Abstract

Physical activity has been shown to impact mental health in in vitro fertilization-embryo transfer (IVF-ET) patients, but the relationship between domain-specific physical activity and mental health in this population remains unclear. In a cross-sectional observational study, 208 patients undergoing IVF-ET with long-term ovulation induction cycles were recruited from a reproductive center. The International Physical Activity Questionnaire and the symptom checklist 90 (SCL-90) were used to assess physical activity levels and mental health status, respectively. Analyses of variance and linear regression analyses were conducted to identify the relationship between physical activity and mental health. There were differences between different physical activity level in times of transfers, years of infertility, and times of abortions. Patients with high levels of physical activity had fewer symptoms than those with low levels of physical activity. Occupation, transport and household physical activity had significant negative correlations with the respective SCL-90 factor scores. Linear regression analysis revealed that occupation physical activity was associated with lower depression and anxiety, and transport physical activity and household physical activity were associated with lower obsessive–compulsive, interpersonal sensitivity, depression, anxiety, and psychoticism scores. The study indicates that increased engagement in physical activity, specifically activities related to occupation, transportation, and household, may be associated with improved mental health among IVF-ET patients.

## Introduction

In recent years, increasing numbers of couples affected by infertility have sought medical help and used assisted reproductive technology (ART) such as in vitro fertilization (IVF) or intracytoplasmic sperm injection to conceive their child. On the other hand, pregnant women are indeed especially susceptible to the development of a mental illness or relapse after the first episode, due to their high-stress levels and lack of social support^1^. Compared with their counterparts who visit the hospital for regular physical examinations, the incidence of anxiety and depression was 15.6% and 21.0% separately in women seeking IVF treatment^2^. Furthermore, compared to women who tried natural conception, women who underwent IVF have greater baseline levels of depressive symptoms and state anxiety, and negative feelings would be maintained over time^3^. It is also noteworthy that as the patient invests more time and effort in preparing for pregnancy, the failure of treatment could incur a heavier psychological burden^4^.

Regular physical activity/exercise is beneficial to an individual’s general health^5^ and reducing the risks of obesity, cardiovascular disease, hypertension, diabetes, osteoporosis, and psychological stress. Exercise during pregnancy has been reported to increase maternal well-being^6^. Despite the evidence of the benefits of exercise in patients’ health, pregnant women tend to reduce physical activity for both physical and psychological reasons^7^. On the other hand, discomfort during exercise, fear of harm to the fetus, and a history of abortion or infertility treatments are common reasons for pregnant women to reduce the amount of their physical activity^8^. We also observed that in clinical practice, following IVF-ET, some patients choose to stop working, reduce their physical activity, and in some cases, even keep a sedentary lifestyle, which would seriously affect the patient’s quality of life^2^. To the best of our knowledge, there has been no relevant research on the relationship between physical activity and the mental health of IVF-ET patients.

Physical activity could be done as a form of transportation, in one’s free time, at work or school, or even while doing household tasks. Since the motivations for participating are likely to differ among these various physical activity domains, the impacts participants experience may also vary. A related meta-analysis has shown that the life domains in which physical activity happens have different influences on the relationship between physical activity and mental health^9^. Leisure-time physical activity and transport physical activity positive associated mental health, while leisure-time physical activity has an inverse association with mental ill-health^9^. Based on previous study, the present study assumes that special domain physical activity associated with the mental health in IVF-ET patients, and physical activity is an independent variable and mental health is a dependent variable. This study aimed to investigate the relationship between domain-specific physical activity and IVF-ET patients’ mental health status. Therefore, the present study filled this gap to improve the mental health and quality of life of pregnant patients following embryo transfer in IVF-ET.

## Materials and methods

### Research subjects

The cross-sectional observational study was undertaken by the reproductive center of Qilu Hospital of Shandong University. Between June 2015 and June 2017, a total of 302 women with IVF-ET long-term ovulation induction cycles were recruited; 37 refused to participate and 57 were screened but deemed ineligible based on criteria. In total, 208 patients were enrolled in the current study. They all had no limitations on physical activity. In ovulation induction, a long gonadotropin-releasing hormone agonist regimen and recombinant human FSH were applied. The maturation of oocytes was stimulated with human chorionic gonadotropin. Intramuscular administration occurs when the anterior guiding follicle reaches an average diameter of 20 mm with two bristles > 16 mm. Oocyte retrieval was conducted after 36 h. Intracytoplasmic sperm injection was conducted according to routine protocols. Women with a positive pregnancy test continue to use vaginal progesterone gels until week 12 of pregnancy.

Inclusion criteria: (1) Patients who received IVF-ET treatment; (2) Who were able to sign an informed consent and were able to cooperate with researchers to complete relevant scale surveys; (3) Revisited Qilu Hospital on time, without treatment by other medical units; (4) Age: < 35 years old; (5) Regular menstrual cycle (27–32 days); (6) IVF-ET treatment using a GnRH-a long COH protocol; (7) The indication of IVF-ET is a tubal factor, and patients with apparent uterine, endocrine and immune infertility are excluded, and male partners are excluded. Exclusion criteria: (1) Patients who have received psychological counselling; (2) Comorbid with other diseases that affect daily activities, such as limb disability, stroke, cardiac insufficiency, etc. (3) Occupations that require high-intensity physical activity, such as food delivery, construction workers, harvest farmers, etc.

### Data collection

A self-compiled general questionnaire was used to collect information about the general information of IVF-ET patients. General information includes age, times of oocyte retrievals, number of pregnancies, number of embryos transferred, number of transfers, years of infertility, times of births, times of abortions, etc. Data were collected by questionnaire. On the day of embryo transfer, prior to providing written informed consent, the patient was thoroughly briefed about the purpose, significance, and content of the questionnaire. Subsequently, on the 7th day following embryo transfer, study participants completed the general questionnaire (e.g. age, education levels, times of oocyte retrievals, number of pregnancies, number of embryos transferred, times of transfers, years of infertility, times of abortions, times of births), IPAQ questionnaire and SCL-90 scale. Following the completion of the questionnaires, statistical analyses were conducted.

### Survey tools

International Physical Activity Questionnaire (IPAQ) was used to evaluate patients’ physical activity after embryo transfer^10,11^. It is recognized as one of the most established and widely used physical activity level questionnaires for adults (15–69 years old) globally, with sufficient validity and reliability^12–14^, IPAQ have been previous applied in the IVF patients^15^. The questionnaire consists of five parts: daily work, housework, transportation, sports and leisure, and sleep. The data collected regarding physical activity included the activity’s intensity, duration, frequency, and surrounding environment. The amount of activity was recorded separately, and each physical activity could be converted into the metabolic equivalent.

IPAQ calculation method^16^: data cleansing and truncation were performed according to the principle of abnormal value elimination. The total amount of weekly physical activity was calculated in (metabolic equivalent) MET-min from the following formula: a particular activity energy expenditure (MET-mins) = the metabolic equivalent value of the activity (MET level) × average daily activity time (mins) × number of weekly activities. The metabolic equivalents of each activity are shown in Table S1, and the MET-min/week was used for the analysis. According to the following principles based on previous study^17^, the total activity level is divided into three levels (high, medium, and low) determined by the intensity of four specific types of activities. High-level physical activity was classified if either of the following two criteria is met: (1) total high-intensity physical activity ≥ 3 days and a total weekly activity level ≥ 1500 MET-min/w; (2) total physical activity ≥ 7 days and a total weekly activity level ≥ 3000 MET-min. Medium-level physical activity was defined as meeting any of the following three criteria: (1) at least 20 min per day of any high-intensity physical activity, total ≥ 3 days; (2) at least 30 min per day of any moderate-intensity physical activity and/or walking activities, total ≥ 5 days; (3) total physical activity ≥ 5 days and a total weekly activity level > 600 MET-min/w. Finally, low-level physical activity was defined by either of the following two criteria: (1) no activity reported; (2) some activity reported but not meeting the above-mentioned medium or high grouping criteria.

The symptom checklist 90 (SCL-90) was compiled by Derogatis et al. of the United States in 1975^18^. It has demonstrated an excellent capability to distinguish people who may be on the verge of psychological disorders. Each item is scored on a 5-point scale (from 1 to 5). There are two leading statistical indicators of SCL-90: (1) Factor score: The scale includes 90 items on a total of 10 factors. Each factor reflects a certain aspect of the subject: somatization, obsessive–compulsive symptoms, interpersonal sensitivity, depression, anxiety, hostility, phobic anxiety, paranoia, psychoticism, and others (reflects sleeping and eating). (2) Global severity index (GSI): Higher GSI scores imply more severe psychopathologic symptoms since they are derived from the mean of all 90 questions in the SCL-90, which is the “global” measure of psychological symptoms.

The stress score was calculated based on previous studies^19,20^, the total score was the sum of the numbers of embryos were transferred, times of transfers, years of infertility, and times of abortions. The higher scores mean more perceived stress in the process of assistant reproductive technology.

### Statistical analysis

The sample size was determined via a priori power analysis using G*Power version 3.1.9.619^21^, and followed the power calculations proposed by Cohen^22^: for a medium regression coefficient (β = 0.2) with a significance level (α) set at 0.05 and a desired statistical power of 80%, the calculated required sample size was 184. Similarly, for a moderate bivariate correlation (r = 0.3) with the same significance level and desired statistical power, the required sample size was 84. Additionally, for an ANOVA with a medium effect size (f = 0.25), the significance level set at 0.05, and a desired statistical power of 80%, the calculated required sample size was 159. For the Chi-square analysis, considering an effect size (f = 0.25), an α error probability of 0.05, power (β error probability) of 0.8, and degrees of freedom equal to 6, the calculated required sample size was 143. Consequently, the sample size in our study should adequately achieve statistical power for our strategy intervention.

All statistical analyses were performed using SPSS software v26.0. Levene’s test was used to assess the homogeneity of variances, and Shapiro–Wilk test was used to evaluated the homogeneity of variances and normality conversion was conducted when needed. Analyses of variance (ANOVA) and Chi-square were conducted to compare clinical data and scores of each factor and GSI of SCL-90 scale among patients with the three levels of physical activity, and the Tukey test was used for post hoc analysis. Pearson’s correlation was used to explore associations between scores of each factor of SCL-90 scale and different intensity and domains of physical activity. Linear regression analysis was performed to investigate the relationship between physical activity and mental health, initially without adjustments and subsequently accounting for covariates. Age, stress scores, and education were identified as potential confounding variables. Covariates associated with mental health, as determined through ANOVA or Pearson's correlation, were included in the adjusted model. Results are given as standardized coefficients (B) with 95% CI. For all tests, two-sided p < 0.05 was considered statically significant.

### Ethics approval and consent to participate

This study was approved by the Ethics Committees of Qilu Hospital of Shandong University (KYLL-2016-331). All participants obtained written informed consent after the procedure was fully explained and before participating in the trial. All procedures contributing to this work follow the ethical standards of the Declaration of Helsinki of 1975, as revised in 2008.

## Results

### Association between physical activity and mental health in IVF-ET-assisted pregnancy patients.

As shown in Table 1, there were 33 (15.8%) IVF-ET-assisted pregnancy patients in the low physical activity level group, 100 (48.1%) in the medium physical activity level group, and 75 (36.1%) in the high physical activity level group. There were differences between different physical activity level in times of transfers, years of infertility, and times of abortions. Comparisons of SCL-90 scores among the three groups of physical activity levels are shown in Table 2. The SCL-90 factor scores such as somatization (*p* = 0.025), obsessive–compulsive (*p* < 0.001), interpersonal sensitivity (*p* = 0.046), depression (*p* < 0.001), anxiety (*p* < 0.001), hostility (*p* < 0.001), phobia anxiety (*p* = 0.014), paranoid ideation (*p* = 0.038), and psychoticism (*p* = 0.005) in the low physical activity level groups were significantly higher than those in the high physical activity level group, and the obsessive–compulsive (*p* = 0.005), interpersonal sensitivity (*p* = 0.008), depression (*p* = 0.002), anxiety (*p* = 0.013), hostility (*p* = 0.032) in medium physical activity level groups were significantly higher than those in the high physical activity level group.

**Table 1:** Comparison of sociodemographic factors and clinical data between different groups of physical activity levels.

Item	Low physical activity level (n = 33)	Medium physical activity level (n = 100)	High physical activity level (n = 75)	*F/*χ^2^	*p* value	Cohen’s *d*/contingency coefficient
Age	28.33 ± 0.45	29.27 ± 0.33	29.29 ± 0.42	1.145	0.320	0.011
Education levels				3.785	0.441	0.134
Junior high school and below	14	39	40			
High school or technical secondary school	11	38	21			
College degree and above	8	23	14			
Times of oocyte retrievals				3.888	0.143	0.135
1	25	73	64			
≥ 2	8	27	11			
Number of pregnancies				1.466	0.845	0.083
0	20	50	39			
1–2	10	41	28			
≥ 3	3	9	8			
Number of embryos transferred				3.872	0.137	0.134
1–2	32	95	75			
≥ 3	1	5	0			
Times of transfers				10.626	0.005	0.220
1–2	22	74	68			
≥ 3	11	26	7			
Years of infertility				22.892	< 0.001	0.315
< 3	12	34	46			
3–5	18	35	20			
> 5	3	31	9			
Times of abortions				7.361	0.021	0.188
0	25	65	62			
≥ 1	9	35	12			
Times of births				4.659	0.096	0.148
0	32	82	57			
≥ 1	2	18	17			
Sedentary time (min)	6383.98 ± 165.29	5824.17 ± 120.79	4776.67 ± 128.20	35.592	< 0.001	0.325
Sleep time (min)	3445.67 ± 152.33	3600.91 ± 81.97	3691.31 ± 63.54	1.356	0.261	0.018

Quantitative data is expressed in mean ± standard error of mean, and qualitative data is expressed in n.

**Table 2:** Comparison of scores of each factor and GSI of SCL-90 scale among the different groups of physical activity levels.

Variables	Low physical activity level (M ± SEM)	Medium physical activity level (M ± SEM)	High physical activity level (M ± SEM)	F	*p*	*p*1 (a vs. c)	*p*2 (a vs. b)	*p*3 (b vs. c)
Somatization	1.51 ± 0.06	1.39 ± 0.04	1.31 ± 0.03*	3.503	0.033	0.025	0.221	0.362
Obsessive–compulsive	1.77 ± 0.07	1.57 ± 0.06	1.34 ± 0.03***^#^	11.250	0.000	0.000	0.072	0.005
Interpersonal sensitivity	1.41 ± 0.07	1.40 ± 0.06	1.17 ± 0.03*^##^	5.240	0.007	0.046	0.999	0.008
Depression	1.50 ± 0.06	1.41 ± 0.05	1.19 ± 0.02***^##^	8.992	0.000	0.001	0.473	0.002
Anxiety	1.55 ± 0.07	1.36 ± 0.05*	1.18 ± 0.02***^#^	10.791	0.000	0.000	0.040	0.013
Hostility	1.59 ± 0.06	1.43 ± 0.05	1.26 ± 0.04***^#^	7.247	0.001	0.001	0.174	0.032
Phobia anxiety	1.31 ± 0.06	1.20 ± 0.04	1.12 ± 0.03*	4.051	0.020	0.014	0.166	0.325
Paranoid ideation	1.28 ± 0.06	1.23 ± 0.04	1.12 ± 0.02*	3.590	0.031	0.038	0.615	0.107
Psychoticism	1.30 ± 0.05	1.20 ± 0.04	1.11 ± 0.02**	5.247	0.006	0.005	0.194	0.122
GSI	1.64 ± 0.09	1.56 ± 0.06	1.53 ± 0.08	0.434	0.649	0.626	0.758	0.943

*GSI* global severity index, ANOVA and turkey post hoc was used in data. M ± SEM: mean ± standard error of mean. *p < 0.05, **p < 0.01, ***p < 0.001 vs. low physical activity level; ^#^*p* < 0.05, ^##^*p* < 0.01 vs. medium physical activity level. The scores of each factor range from 0 to 5 points.

### Association between total and domains of physical activity and mental health in IVF-ET-assisted pregnancy patients.

The present study further explored the relationships between mental health and different domains of physical activity using correlation analysis (Table 3). Total physical activity had significant negative correlations with the somatization (*p* = 0.007), obsessive–compulsive (*p* < 0.001), interpersonal sensitivity (*p* = 0.003), depression (*p* < 0.001), anxiety (*p* < 0.001), hostility (*p* = 0.004), phobia anxiety (*p* = 0.025), psychoticism (*p* = 0.007). Occupation activity was correlated with depression (*p* = 0.038) and anxiety (*p* = 0.043), and transport activity was correlated with obsessive–compulsive (*p* = 0.035), interpersonal sensitivity (*p* = 0.024), depression (*p* = 0.004), anxiety (*p* = 0.023), phobia anxiety (*p* = 0.041), and psychoticism (*p* = 0.019) of SCL-90, while household activity was associated with obsessive–compulsive (*p* = 0.014), interpersonal sensitivity (*p* = 0.031), depression (*p* = 0.020), anxiety (*p* = 0.048) and psychoticism (*p* = 0.01) (Table 3).

**Table 3:** Correlation among scores of each factor and GSI of SCL-90 scale and total and different domains of physical activity.

Variables	Total physical activity	Occupation physical activity	Transport physical activity	Household physical activity	Recreational physical activity
Somatization	− 0.237**	− 0.114	− 0.160	− 0.061	− 0.145
Obsessive–compulsive	− 0.355***	− 0.141	− 0.211*	− 0.244*	− 0.095
Interpersonal sensitivity	− 0.260**	− 0.144	− 0.226*	− 0.215*	0.019
Depression	− 0.341***	− 0.209*	− 0.285**	− 0.234*	− 0.076
Anxiety	− 0.343***	− 0.203*	− 0.227*	− 0.198*	− 0.129
Hostility	− 0.252**	− 0.112	− 0.153	− 0.145	− 0.085
Phobia anxiety	− 0.200*	− 0.052	− 0.205*	− 0.138	− 0.094
Paranoid ideation	− 0.169	− 0.122	− 0.174	− 0.145	− 0.052
Psychoticism	− 0.239**	− 0.154	− 0.234*	− 0.257*	0.064
GSI	− 0.084	− 0.025	− 0.029	− 0.087	− 0.137

*GSI* global severity index. *p < 0.05, *p < 0.01, ***p < 0.001 of the Pearson correlation coefficient. The MET-min/week of physical activity was used for the analysis.

### Regression analysis between physical activity and mental health in IVF-ET patients.

Linear regression analyses showed that the unadjusted association of total physical activity and somatization (β = − 0.230, *p* = 0.007), obsessive–compulsive (β = − 0.345, *p* < 0.001), interpersonal sensitivity (β = − 0.253, *p* = 0.003), depression (β = − 0.328, *p* < 0.001), anxiety (β = − 0.331, *p* < 0.001), hostility (β = − 0.242, *p* = 0.004), phobia anxiety (β = − 0.189, *p* = 0.025), psychoticism (β = − 0.230, *p* = 0.007) were significant (Table 4). When age, stress scores, and education were added to the model, the association of total physical activity with the mental health remained statistically significant (Table 4).

**Table 4:** Associations between total physical activity and factor scores of SCL-90 in IVF patients.

Variables	R^2^	Standardized β coefficient	95% CI		*p* value
Somatization					
Unadjusted regression model	0.237				
Total physical activity		− 0.230	− 0.397	− 0.062	0.007
Adjusted regression model	0.266				
Total physical activity		− 0.198	− 0.376	− 0.020	0.030
Stress scores		0.087	− 0.089	0.262	0.331
Age		0.052	− 0.124	0.228	0.561
Education		0.070	− 0.104	0.243	0.430
Obsessive–compulsive					
Unadjusted regression model	0.355				
Total physical activity		− 0.345	− 0.506	− 0.183	< 0.001
Adjusted regression model	0.432				
Total physical activity		− 0.350	− 0.518	− 0.183	< 0.001
Stress scores		− 0.101	− 0.265	0.064	0.227
Age		0.062	− 0.103	0.227	0.460
Education		0.208	0.045	0.371	0.013
Interpersonal sensitivity					
Unadjusted regression model	0.260				
Total physical activity		− 0.253	− 0.419	− 0.086	0.003
Adjusted regression model	0.331				
Total physical activity		− 0.262	− 0.437	− 0.087	0.004
Stress scores		− 0.099	− 0.271	0.073	0.255
Age		0.030	− 0.143	0.203	0.729
Education		0.169	− 0.002	0.339	0.053
Depression					
Unadjusted regression model	0.341				
Total physical activity		− 0.328	− 0.490	− 0.167	< 0.001
Adjusted regression model	0.378				
Total physical activity		− 0.357	− 0.528	− 0.186	< 0.001
Stress scores		− 0.125	− 0.294	0.045	0.149
Age		0.057	− 0.112	0.226	0.506
Education		0.082	− 0.085	0.250	0.332
Anxiety					
Unadjusted regression model	0.342				
Total physical activity		− 0.331	− 0.492	− 0.170	< 0.001
Adjusted regression model	0.399				
Total physical activity		− 0.334	− 0.503	− 0.165	< 0.001
Stress scores		− 0.087	− 0.254	0.079	0.299
Age		0.001	− 0.166	0.168	0.989
Education		0.175	0.011	0.340	0.037
Hostility					
Unadjusted regression model	0.252				
Total physical activity		− 0.242	− 0.408	− 0.077	0.004
Adjusted regression model	0.297				
Total physical activity		− 0.212	− 0.388	− 0.037	0.018
Stress scores		0.029	− 0.143	0.202	0.737
Age		− 0.036	− 0.209	0.138	0.683
Education		0.152	− 0.019	0.323	0.080
Phobia anxiety					
Unadjusted regression model	0.200				
Total physical activity		− 0.189	− 0.354	− 0.024	0.025
Adjusted regression model	0.271				
Total physical activity		− 0.186	− 0.360	− 0.012	0.037
Stress scores		− 0.059	− 0.231	0.112	0.495
Age		− 0.124	− 0.296	0.049	0.158
Education		0.110	− 0.060	0.280	0.204
Psychoticism					
Unadjusted regression model	0.239				
Total physical activity		− 0.230	− 0.396	− 0.064	0.007
Adjusted regression model	0.314				
Total physical activity		− 0.258	− 0.433	− 0.084	0.004
Stress scores		− 0.149	− 0.321	0.022	0.087
Age		− 0.080	− 0.253	0.092	0.358
Education		0.091	− 0.079	0.261	0.292

*CI* confidence interval. The MET-min/week of physical activity was used for the analysis.

Linear regression analyses showed that the unadjusted association of occupation activity and depression (β = − 0.199, *p* = 0.038), anxiety (β = − 0.188, *p* = 0.043) were significant; transport physical activity and obsessive–compulsive (β = − 0.200, *p* = 0.035), interpersonal sensitivity (β = − 0.219, *p* = 0.024), depression (β = − 0.267, *p* = 0.004), anxiety (β = − 0.207, *p* = 0.023), phobia anxiety (β = − 0.203, *p* = 0.041) and psychoticism (β = − 0.231, *p* = 0.019) scores were significant; household physical activity and obsessive–compulsive (β = − 0.252, *p* = 0.014), interpersonal sensitivity (β = − 0.227, *p* = 0.031), depression (β = − 0.238, *p* = 0.020), anxiety (β = − 0.196, *p* = 0.048), and psychoticism (β = − 0.276, *p* = 0.010) scores were significant (Table S2).

## Discussion

At present, the reproductive center of the hospital uses the IVF-ET long program to stimulate the ovulation cycle to help infertile patients be pregnant. At the same time, bed rest after embryo transfer is still routine practice in some reproductive centers, and it is believed that reducing postoperative activity can help improve pregnancy outcomes. Walker et al. believe that, in most cases, physical activity during pregnancy is beneficial to both mother and fetus^23^; it is also applicable to patients who are pregnant after IVF-ET. So far, there is still a lack of quantitative data on relationship between physical activity and mental health after an embryo transfer in IVF-ET patients.

The previous study has shown that there were elevated symptoms of anxiety and depression among pregnant individuals^24^, which may have long-term impacts on their children^25^. In addition, exercise during pregnancy could reduce the rate of occurrence of antenatal depression and depressive symptoms^26,27^. What’s more, high levels of physical activity during pregnancy decreased the odds of developing prenatal depression and anxiety^28^. Consistent with the previous study, the present study found that there were statistically significant associations of different physical activity levels with mental health in IVF-ET patients. In addition, patients with high levels of activity demonstrated significantly greater differences mental health compared to their counterparts with low levels of activity, in contrast, the distinctions between those with moderate-level activity and low-level activity were less prominent. Extra bed rest may cause excessive psychological stress and adversely affect pregnancy outcomes. While our study findings suggest a positive association between physical activity and the mental health of IVF-ET patients, future prospective studies incorporating interventions are warranted to further explore the potential benefits of physical activity on the mental health outcomes in IVF-ET patients.

The relationship between mental health and physical activity is inconsistent across different life domains, and it is critical to explore the context that may influence the relationship between physical activity and mental health. The previous meta-analysis showed that leisure-time physical activity and transport physical activity were positively associated with mental health^9^. However, it should be noted that even if the amount of activity is “high” in this study, there is no too strenuous exercise, let alone those who do physical exercise. In this study, 21.63% of patients continued working following embryo transfer, and only 7.21% underwent work-related heavy physical activity, such as going up and down stairs every day. There were 12.72% of patients who were engaged in heavy physical housework daily, such as mopping the floor in a short period. However, 15.87% of patients with low-level activity did not engage in any housework and activities, instead, they were basically in a recumbent state. It is notable from the present study that IVF-ET patients may perform a limited number of physical activities, therefore, it is imperative for more related propaganda and education during the assisted reproductive process.

Previous studies have indicated that anxiety and depression in women with infertility are affected by multiple factors such as family habitation^29^, smoking and drinking status of their partners^30^, family economic status^31^, and their age^32^ and educational level^33^. The present results did not observe a relationship between age and perceived stress with mental health, which may be because of the status of the investigated groups of IVF-ET patients who have relatively good mental health. However, the results showed that total activity especially occupation, transport, and household activities correlated with variables of SCL-90. Transport positively associated with mental health possibly supported this phenomenon that those who walk or bike to work experienced enjoyment^9^. Although the previous study has indicated that household activity had no relationship with mental health or mental disorders^9^, the present study found the results opposite with previous. In fact, physical activity in home isolation during the epidemic helped improve adults’ depression, and anxiety, and increased sleep duration^34^. Sedentary behaviors such as television time have been connected with psychological anguish^35^, whereas lowering television viewing may contribute to reduced psychological distress, particularly in women^36^. Increasing activity during labor is not likely to be worthwhile in reducing the prevalence of mental disorders, which indicated the work-related physical activity was positively associated with mental ill-health^9,37^, as individuals with occupations that involve higher amounts of physical activity are more likely to experience more work and stress. The present study found the occupational physical activity was positive with mental health, which may related the nature of work, access to greenspace, job satisfaction^37^. In addition, no correlation between the leisure physical activity could due to the women in the study may just have done little PA during leisure time within the past 7 days. Diverse domains of physical activity exhibit distinct correlations with post-exercise mood. Consequently, forthcoming research endeavors should delve into the nuanced, long-term relationships between domain-specific physical activity and mental health outcomes.

This study has significant theoretical value for clarifying the correlation between physical activity and mental health in IVF patients, which provides the basis for improving mental health in the IVF-ET process. On the other hand, this study has limitations as well, including a relatively small sample size and limited data collected at baseline. As the present was cross-sectional observational study, there was no criteria and data determined regarding the type of physical activity prior to the start of the study. Furthermore, the study was cross-sectional and could not establish a causal relationship. In addition, the relevant confounding variables were not collected in present study, which may have resulted in erroneous associations between physical activity and these variables, which could affect the generalizability of the conclusion. Furthermore, the use of the IPAQ which assesses physical activity over the past 7 days, introduces a potential overlap with laboratory visits, possibly not fully capturing participants' habitual or typical physical activity patterns.

## Conclusion

This study showed that total and domains-specific physical activity after embryo transfer may be associated with mental health in IVF patients. In particular, appropriate occupation activity, transport activity and household activity could be implemented for IVF-ET patients which may beneficial for their mental health. The stakeholder should think highly of the domain-specific physical activity of the IVF patients in developing interventions, and treatment programs in the future.

### Supplementary Information

Supplementary Table S1.

Supplementary Table S2.

## Data Availability

The datasets generated during and/or analysed during the current study are available from the corresponding author on reasonable request.

## Abbreviations

ANOVA: Analyses of variance
ART: Assisted reproductive technology
GSI: Global severity index
IPAQ: International Physical Activity Questionnaire
IVF-ET: In vitro fertilization-embryo transfer
MET: Metabolic equivalent
SCL-90: The symptom checklist 90
