# Supplementary material for: Association between domain-specific physical activity and mental health status after embryo transfer in IVF-ET-assisted pregnancy patients
Source: Sci Rep. 2024 Feb 28;14:4928. doi: 10.1038/s41598-024-55097-3 (PMC10902343; doi:10.1038/s41598-024-55097-3)
Supplement: Supplementary file 1 — Supplementary Table S1. [file 41598_2024_55097_MOESM1_ESM.docx]

Table S1 Types of physical activities in International Physical Activity Questionnaire and Metabolic equivalent value

| Types of PA | Project of PA | Intensity of PA | Metabolic  equivalent |
| --- | --- | --- | --- |
| Occupation Activity | Walking | Low | 3.3 |
|  | Moderate intensity | Moderate | 4.0 |
|  | High intensity | High | 8.0 |
| Transport Activity | Walking | Low | 3.3 |
|  | Biking | Moderate | 6.0 |
| Household Activity | Moderate intensity indoor household | Moderate | 3.0 |
|  | Moderate intensity outdoor household | Moderate | 4.0 |
|  | High intensity outdoor household | High | 5.5 |
| Recreational Activity | Walking | Low | 3.3 |
|  | Moderate intensity | Moderate | 4.0 |
|  | High intensity | High | 8.0 |
