# Supplementary material for: Association between domain-specific physical activity and mental health status after embryo transfer in IVF-ET-assisted pregnancy patients
Source: Sci Rep. 2024 Feb 28;14:4928. doi: 10.1038/s41598-024-55097-3 (PMC10902343; doi:10.1038/s41598-024-55097-3)
Supplement: Supplementary file 2 — Supplementary Table S2. [file 41598_2024_55097_MOESM2_ESM.docx]

Table S2. Associations between domains of physical activity and factor scores of SCL-90 in IVF Patients.

| **Variables** | **R^2^** | **Standardized**  **β coefficient** | **95% CI** | | ***p***  **value** |
| --- | --- | --- | --- | --- | --- |
| **Occupation Activity** |  |  |  | |  |
| Depression |  |  |  |  |  |
| Unadjusted regression model | 0.209 |  |  |  |  |
| Total physical activity |  | -0.199 | -0.387 | -0.012 | 0.038 |
| Adjusted regression model | 0.257 |  |  |  |  |
| Total physical activity |  | -0.217 | -0.407 | -0.026 | 0.026 |
| Stress scores |  | 0.012 | -0.192 | 0.216 | 0.908 |
| Age |  | 0.054 | -0.150 | 0.259 | 0.599 |
| Education |  | 0.135 | -0.064 | 0.334 | 0.181 |
| Anxiety |  |  |  |  |  |
| Unadjusted regression model | 0.203 |  |  |  |  |
| Total physical activity |  | -0.188 | -0.370 | -0.006 | 0.043 |
| Adjusted regression model | 0.287 |  |  |  |  |
| Total physical activity |  | -0.202 | -0.384 | -0.019 | 0.031 |
| Stress scores |  | 0.074 | -0.120 | 0.268 | 0.450 |
| Age |  | -0.042 | -0.238 | 0.153 | 0.669 |
| Education |  | 0.181 | -0.009 | 0.372 | 0.061 |
| **Transport Activity** |  |  |  |  |  |
| Obsessive-compulsive |  |  |  |  |  |
| Unadjusted regression model | 0.211 |  |  |  |  |
| Total physical activity |  | -0.200 | -0.386 | -0.014 | 0.035 |
| Adjusted regression model | 0.323 |  |  |  |  |
| Total physical activity |  | -0.196 | -0.382 | -0.009 | 0.040 |
| Stress scores |  | -0.070 | -0.271 | 0.132 | 0.495 |
| Age |  | 0.065 | -0.137 | 0.267 | 0.524 |
| Education |  | 0.228 | 0.032 | 0.423 | 0.023 |
| Interpersonal sensitivity |  |  |  |  |  |
| Unadjusted regression model | 0.226 |  |  |  |  |
| Total physical activity |  | -0.219 | -0.409 | -0.030 | 0.024 |
| Adjusted regression model | 0.282 |  |  |  |  |
| Total physical activity |  | -0.222 | -0.416 | -0.029 | 0.025 |
| Stress scores |  | -0.085 | -0.294 | 0.124 | 0.420 |
| Age |  | 0.046 | -0.163 | 0.255 | 0.661 |
| Education |  | 0.149 | -0.054 | 0.351 | 0.149 |
| Depression |  |  |  |  |  |
| Unadjusted regression model | 0.285 |  |  |  |  |
| Total physical activity |  | -0.267 | -0.448 | -0.086 | 0.004 |
| Adjusted regression model | 0.306 |  |  |  |  |
| Total physical activity |  | -0.268 | -0.455 | -0.081 | 0.005 |
| Stress scores |  | -0.029 | -0.233 | 0.174 | 0.775 |
| Age |  | 0.062 | -0.140 | 0.263 | 0.545 |
| Education |  | 0.086 | -0.110 | 0.282 | 0.384 |
| Anxiety |  |  |  |  |  |
| Unadjusted regression model | 0.227 |  |  |  |  |
| Total physical activity |  | -0.207 | -0.385 | -0.029 | 0.023 |
| Adjusted regression model | 0.276 |  |  |  |  |
| Total physical activity |  | -0.186 | -0.368 | -0.004 | 0.045 |
| Stress scores |  | 0.049 | -0.148 | 0.246 | 0.623 |
| Age |  | -0.041 | -0.238 | 0.155 | 0.679 |
| Education |  | 0.143 | -0.047 | 0.334 | 0.139 |
| Phobia anxiety |  |  |  |  |  |
| Unadjusted regression model | 0.205 |  |  |  |  |
| Total physical activity |  | -0.203 | -0.398 | -0.009 | 0.041 |
| Adjusted regression model | 0.270 |  |  |  |  |
| Total physical activity |  | -0.187 | -0.385 | 0.011 | 0.064 |
| Stress scores |  | -0.023 | -0.237 | 0.192 | 0.834 |
| Age |  | -0.122 | -0.336 | 0.093 | 0.263 |
| Education |  | 0.148 | -0.059 | 0.356 | 0.160 |
| Psychoticism |  |  |  |  |  |
| Unadjusted regression model | 0.234 |  |  |  |  |
| Total physical activity |  | -0.231 | -0.424 | -0.038 | 0.019 |
| Adjusted regression model | 0.300 |  |  |  |  |
| Total physical activity |  | -0.240 | -0.435 | -0.044 | 0.017 |
| Stress scores |  | -0.153 | -0.365 | 0.059 | 0.154 |
| Age |  | -0.055 | -0.266 | 0.157 | 0.610 |
| Education |  | 0.118 | -0.087 | 0.323 | 0.257 |
| **Household Activity** |  |  |  |  |  |
| Obsessive-compulsive |  |  |  |  |  |
| Unadjusted regression model | 0.244 |  |  |  |  |
| Total physical activity |  | -0.252 | -0.452 | -0.051 | 0.014 |
| Adjusted regression model | 0.338 |  |  |  |  |
| Total physical activity |  | -0.240 | -0.444 | -0.035 | 0.022 |
| Stress scores |  | -0.058 | -0.257 | 0.141 | 0.567 |
| Age |  | 0.092 | -0.112 | 0.295 | 0.374 |
| Education |  | 0.208 | 0.011 | 0.404 | 0.038 |
| Interpersonal sensitivity |  |  |  |  |  |
| Unadjusted regression model | 0.215 |  |  |  |  |
| Total physical activity |  | -0.227 | -0.434 | -0.021 | 0.031 |
| Adjusted regression model | 0.266 |  |  |  |  |
| Total physical activity |  | -0.223 | -0.437 | -0.009 | 0.042 |
| Stress scores |  | -0.067 | -0.276 | 0.141 | 0.524 |
| Age |  | 0.067 | -0.146 | 0.280 | 0.535 |
| Education |  | 0.134 | -0.072 | 0.339 | 0.201 |
| Depression |  |  |  |  |  |
| Unadjusted regression model | 0.234 |  |  |  |  |
| Total physical activity |  | -0.238 | -0.437 | -0.039 | 0.020 |
| Adjusted regression model | 0.260 |  |  |  |  |
| Total physical activity |  | -0.241 | -0.449 | -0.033 | 0.024 |
| Stress scores |  | -0.002 | -0.207 | 0.202 | 0.982 |
| Age |  | 0.082 | -0.126 | 0.289 | 0.435 |
| Education |  | 0.074 | -0.126 | 0.275 | 0.463 |
| Anxiety |  |  |  |  |  |
| Unadjusted regression model | 0.198 |  |  |  |  |
| Total physical activity |  | -0.196 | -0.390 | -0.001 | 0.048 |
| Adjusted regression model | 0.250 |  |  |  |  |
| Total physical activity |  | -0.166 | -0.368 | 0.036 | 0.106 |
| Stress scores |  | 0.066 | -0.130 | 0.262 | 0.507 |
| Age |  | -0.028 | -0.229 | 0.173 | 0.783 |
| Education |  | 0.134 | -0.060 | 0.328 | 0.173 |
| Psychoticism |  |  |  |  |  |
| Unadjusted regression model | 0.257 |  |  |  |  |
| Total physical activity |  | -0.276 | -0.484 | -0.068 | 0.010 |
| Adjusted regression model | 0.302 |  |  |  |  |
| Total physical activity |  | -0.266 | -0.482 | -0.051 | 0.016 |
| Stress scores |  | -0.136 | -0.346 | 0.074 | 0.201 |
| Age |  | -0.027 | -0.242 | 0.188 | 0.801 |
| Education |  | 0.098 | -0.110 | 0.305 | 0.352 |

CI: Confidence interval. The MET-min/week of physical activity was used for the analysis.
